# Supplementary material for: DPPA as a Potential Cell Membrane Component Responsible for Binding Amyloidogenic Protein Human Cystatin C
Source: Molecules. 2024 Jul 23;29(15):3446. doi: 10.3390/molecules29153446 (PMC11313537; doi:10.3390/molecules29153446)
Supplement: Supplementary file 1 [file molecules-29-03446-s001.zip › molecules-3051747-supplementary.pdf]

DPPA as a potential cell membrane component responsible for binding amyloidogenic protein human cystatin C

Igor Zhukov [1], Emilia Sikorska [2], Marta Orlikowska [3], Magdalena Górniewicz-Lorens [4,5], Mariusz Kepczynski [4], Paulina Czaplewska [6], Przemyslaw Jurczak [6,7]*

[1] Biological NMR Facility, Institute of Biochemistry and Bioscience, Polish Academy of Science, Warsaw, Poland

[2] Department of Organic Chemistry, Faculty of Chemistry, University of Gdansk, Gdansk, Poland

[3] Department of Biomedical Chemistry, Faculty of Chemistry, University of Gdansk, Gdansk, Poland

[4] Faculty of Chemistry, Jagiellonian University, Krakow, Poland

[5] Doctoral School of Exact and Natural Sciences, Jagiellonian University, Krakow, Poland

[6] Laboratory of Mass Spectrometry, Intercollegiate Faculty of Biotechnology UG&MUG, University of Gdansk, Gdansk, Poland

[7] Biomacromolecule Research Team, RIKEN Center for Sustainable Resource Science, Wako-shi, Saitama, Japan

* Corresponding author: Przemyslaw Jurczak, +48 58 523 5397, przemyslaw.jurczak@ug.edu.pl

Supporting Information

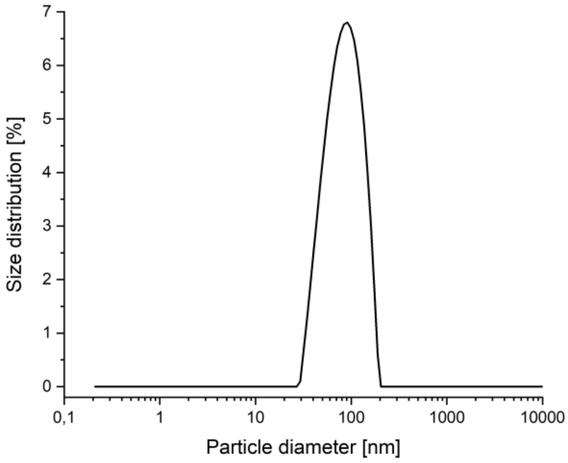

Figure S1. The DLS curve registered for DPPA liposome solution (0.1 mg/mL) at 25°C. Results hydrophobic diameter: 81.92 nm, polydispersity index: 21.9%, transmittance: 85.7%.

Deleted: –

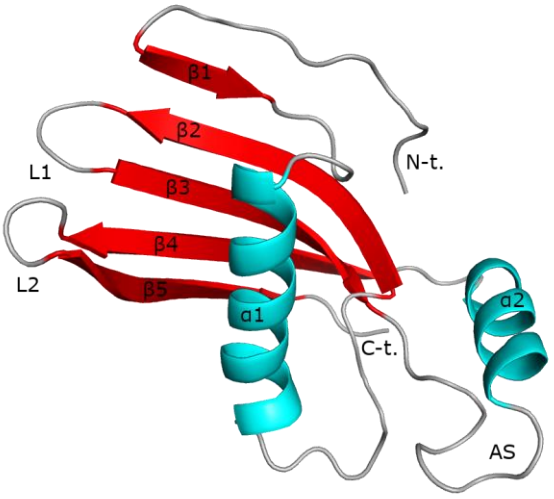

Figure S2. The structure of hCC V57G protein with the secondary structure elements marked (PDB 6RPV).

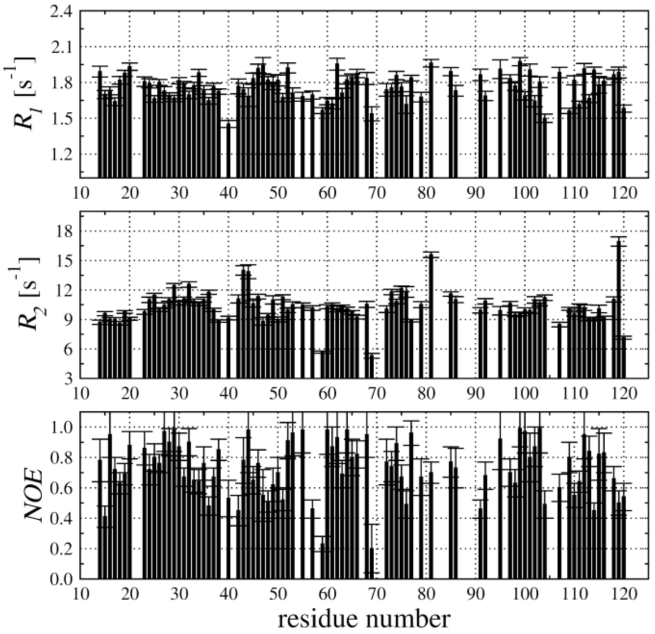

Figure S3. ^{15}N relaxation data (R_1, R_2, and ^{1}H-^{15}N NOE) obtained at magnetic field 11.7 T and 298 K for hCC V57G in the presence of DPPA phospholipid liposomes.

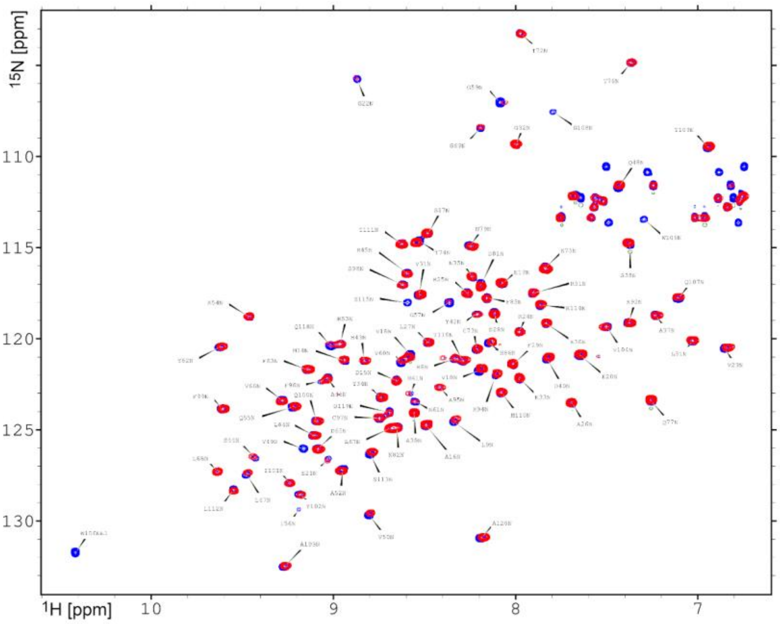

Figure S4. Overlay of the ^{1}H-^{15}N HSQC spectra acquired for the hCC V57G protein in the presence of DPPA phospholipids before (blue) and after (red) the addition of $Cr(AcAc)_3$.

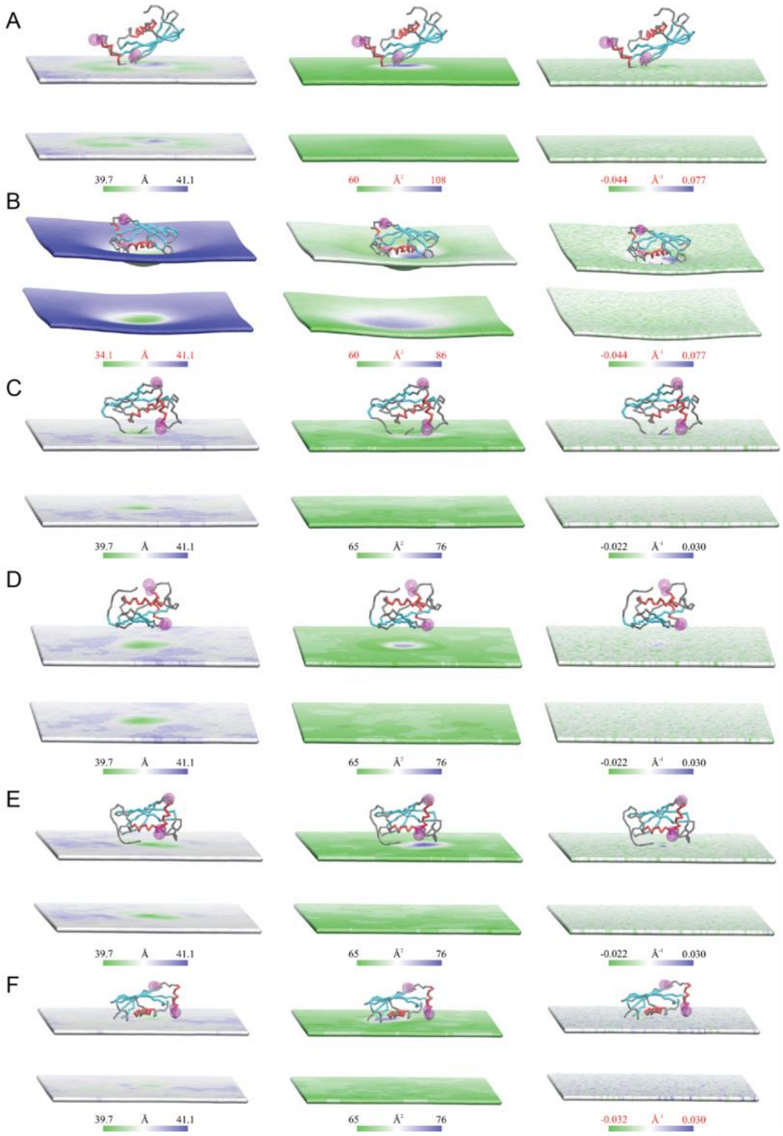

Figure S5. Local properties of the DPPA bilayer in the systems with hCC V57G protein. The time-averaged local bilayer thickness, area per lipids (APL), and mean curvature of the DPPA membrane are shown in the left, middle, and right panels respectively. Panels A–F correspond to models I–VI, respectively. The data were averaged during the last 500 ns of the DPPA binding CG MD simulations for the hCC V57G protein. Phosphate beads of the lipid headgroups were considered for calculations, and 100 bins were used along x and y axes. Different scales are marked in red.

Deleted: time averaged

Deleted: -

Deleted: the

Deleted: -

Deleted: was

Table S1. Composition of the DPPA phospholipid bilayer systems.

Model	Lipid types		Ion types		Protein	Water	Initial system size [x, y, z; Å]	Total simulation time [μs]
	Outer	Inner	Na^+	Cl^-				
I	200 DPPA	200 DPPA	562	165	1	9,855	110,110,152	5
II	200 DPPA	200 DPPA	516	119	1	10,272	110,110,148	5
No protein	200 DPPA	200 DPPA	652	252	-	17,819	108,108,240	1
III	200 DPPA	200 DPPA	652	255	1	17,551	113,113,209	5
IV	200 DPPA	200 DPPA	652	255	1	17,599	113,113,209	5
V	200 DPPA	200 DPPA	652	255	1	17,300	113,113,209	5
VI	200 DPPA	200 DPPA	652	255	1	17,740	113,113,209	5
